# Unravelling the effect of two herbicide resistance mutations on acetolactate synthase kinetics and growth traits

**DOI:** 10.1093/jxb/eraa120

**Published:** 2020-03-09

**Authors:** Ning Zhao, Yanyan Yan, Long Du, Xiaolin Zhang, Weitang Liu, Jinxin Wang

**Affiliations:** 1 College of Plant Protection, Shandong Agricultural University, Tai’an, China; 2 Key Laboratory of Pesticide Toxicology and Application Technology, Shandong Agricultural University, Tai’an, China; 3 State Key Laboratory of Crop Biology, College of Horticulture Science and Engineering, Shandong Agricultural University, Tai’an, China; 4 Pest Bio-control Lab, Shandong Peanut Research Institute, Qingdao, China; 5 Cardiff University, UK

**Keywords:** *Alopecurus aequalis*, ALS kinetics, gene mutation, grass weed, growth competition, resistance cost/advantage

## Abstract

Gene mutations conferring herbicide resistance are hypothesized to have negative pleiotropic effects on plant growth and fitness, which may in turn determine the evolutionary dynamics of herbicide resistance alleles. We used the widespread, annual, diploid grass weed *Alopecurus aequalis* as a model species to investigate the effect of two resistance mutations—the rare Pro-197-Tyr mutation and the most common mutation, Trp-574-Leu—on acetolactate synthase (ALS) functionality and plant growth. We characterized the enzyme kinetics of ALS from two purified *A. aequalis* populations, each homozygous for the resistance mutation 197-Tyr or 574-Leu, and assessed the pleiotropic effects of these mutations on plant growth. Both mutations reduced sensitivity of ALS to ALS-inhibiting herbicides without significant changes in extractable ALS activity. The 197-Tyr mutation slightly decreased the substrate affinity (corresponding to an increased *K*_m_ for pyruvate) and maximum reaction velocity (*V*_max_) of ALS, whereas the 574-Leu mutation significantly increased these kinetics. Significant decrease or increase in plant growth associated, respectively, with the 197-Tyr and 574-Leu resistance mutations was highly correlated with their impact on ALS kinetics, suggesting more likely persistence of the 574-Leu mutation than the 197-Tyr mutation if herbicide application is discontinued.

## Teaser

The herbicide resistance mutations Pro-197-Tyr and Trp-574-Leu have contrary effects on both pyruvate substrate binding and maximum reaction velocity of acetolactate synthase, highly correlated with the cost of resistance.

## Introduction

Acetolactate synthase (ALS; EC 2.2.1.6), also known as acetohydroxyacid synthase (AHAS), is a key plant enzyme catalysing the first step in the biosynthesis of the branched-chain amino acids: valine, leucine, and isoleucine (Duggleby and Pang, 2000; Zhou *et al.*, 2007). ALS isozymes are the common target of five chemical classes: sulfonylurea, imidazolinone, triazolopyrimidine, pyrimidinyl-thiobenzoate, and sulfonyl-aminocarbonyl-triazolinone (Duggleby *et al.*, 2008). However, ALS inhibitors are susceptible to the evolution of resistance, and as many as 162 weed species worldwide show resistant phenotypes (Heap, 2019). Target-site resistance (TSR) is the most common mechanism resulting in resistance to an ALS inhibitor (Yu and Powles, 2014), and at least 28 resistance-endowing gene mutations affecting eight conserved amino acid residues in the ALS enzyme have been identified in weed biotypes with field-evolved resistance (Tranel *et al.*, 2019). Codons 197 and 574 of ALS are by far the two most commonly identified mutated positions (Powles and Yu, 2010; Tranel *et al.*, 2019).

Gene mutations endowing weed plants with TSR allow these weeds to survive herbicide treatments. However, some mutations confer adverse pleiotropic effects on plant growth and fitness by changing enzyme functionality, resulting in insufficient or excessive product biosynthesis (Vila-Aiub et al., 2009*b*, 2015*b*). Mutations in genes encoding both 5-enolpyruvylshikimate-3-phosphate synthase and acetyl-CoA carboxylase (ACCase) can reduce activity of the corresponding enzymes (Healy-Fried *et al.*, 2007; Yu *et al.*, 2007; Vila-Aiub *et al.*, 2015*b*). However, the situation in ALS is more complex, with specific amino acid substitutions causing reduced, increased, or unchanged enzyme activity in different weed species (Boutsalis *et al.*, 1999; Preston *et al.*, 2006; Ashigh and Tardif, 2007; Yu *et al.*, 2010; Vercellino *et al.*, 2018; Yang *et al.*, 2018). A case-by-case analysis is thus required to investigate the precise impact of specific gene modifications on ALS kinetics and the fitness of resistant versus susceptible plants.

Shortawn foxtail (*Alopecurus aequalis* Sobol.), an annual diploid species, is native to North America but has invaded other regions throughout Europe and temperate Asia (Cope, 1982). In China, it is an invasive grass weed severely infesting wheat and canola fields, causing significant yield reduction (Zhao *et al.*, 2018). As a result of long-term chemical control, this species has evolved both TSR and non-target-site-based resistance (NTSR) to herbicides with different modes of action (Zhao et al., 2017, 2019). Mutations at two ALS (Pro-197 and Trp-574) codons in *A. aequalis* plants with TSR are known to impact the efficacy of ALS inhibitors (reviewed in Zhao *et al.*, 2019). Interestingly, we previously found that two mutations, Pro-197-Tyr and Trp-574-Leu, are by far the rarest and the most common mutations, respectively, in *A. aequalis* plants exhibiting TSR to ALS herbicides (Guo *et al.*, 2018; Zhao *et al.*, 2019). Here, we generated *A. aequalis* populations with all individuals homozygous either for the resistance mutation 197-Tyr or for 574-Leu. Using these genetically well-characterized plants, we determined the effect of these two mutations on ALS functionality and plant growth by comparing ALS kinetics and fitness-related growth traits with those of wild-type ALS, herbicide-susceptible populations. This study provides a better understanding of evolutionary preference among ALS herbicide resistance alleles in *A. aequalis* by considering their pleiotropic effects on plant growth and fitness.

## Materials and methods

### Plant material

**Fig. 1.**
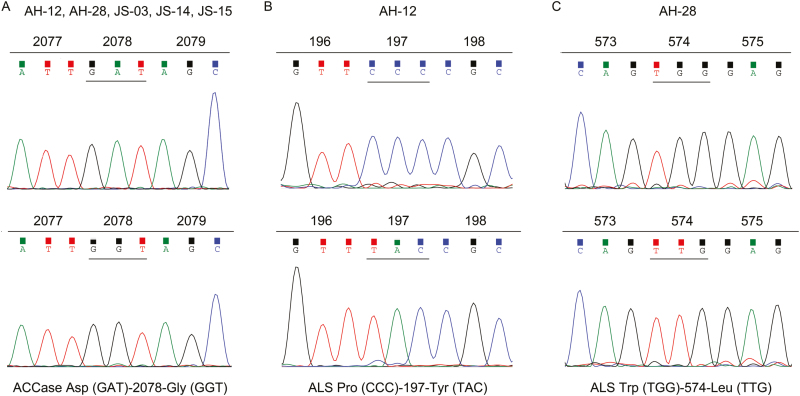
Typical sequence diagrams for purified *A. aequalis* subpopulations. All populations harbor a homozygous mutation in the *ACCase* gene resulting in Asp-2078-Gly mutation (A). Populations AH-12 and AH-28 with homozygous mutations in their *ALS1* genes resulting in Pro-197-Tyr (B) and Trp-574-Leu (C) mutations, respectively. Populations JS-03, JS-14, and JS-15 possess *ALS* genes encoding wild-type 197-Pro.

From May 2015 to May 2018, field-evolved *A. aequalis* populations with suspected resistance to ALS inhibitors were collected across the provinces of Jiangsu (JS), Anhui (AH), Henan, and Shandong, China. After primary single-dose testing and detailed molecular characterization, resistant populations were identified and their resistance mechanisms were investigated (unpublished data). To eliminate the potential impacts of other resistance mechanisms on fitness-related growth traits, two populations (designated AH-12 and AH-28) with both *ACCase* and *ALS* gene mutations were selected, and their resistance to fenoxaprop-*P*-ethyl and mesosulfuron-methyl (MM) was determined to be specifically due to the particular gene mutations. AH-12 and AH-28 carried the same homozygous mutation in their *ACCase* genes resulting in Asp-2078-Gly substitution (Fig. 1A), while mutations causing 197-Tyr and 574-Leu substitutions were respectively located in their *ALS* genes. Purified subpopulations of *A. aequalis* plants homozygous (RR) for the specific ALS herbicide resistance mutations 197-Tyr (Fig. 1B) and 574-Leu (Fig. 1C) were generated by self-pollination. Two *ALS* genes (*ALS1* and *ALS2*) were identified in all purified *A. aequalis* subpopulations with only *ALS1* mutated in all resistant populations. Wild-type *ALS2* was the same in all resistant plants (data not shown). Homozygosity of progeny plants for the specific *ALS* gene mutation in each purified population was reconfirmed as described by Zhao *et al.* (2019). These two purified resistant populations were used in all subsequent experiments.

Three MM-susceptible *A. aequalis* populations (designated JS-03, JS-14, and JS-15, hereinafter called S1, S2, and S3) harboring both the homozygous *ACCase* gene mutation resulting in Asp-2078-Gly substitution and the susceptible *ALS* gene were used as ALS-wild-type controls (Fig. 1A). Herbicide sensitivities in these three *A. aequalis* populations were also compared in the presence of piperonyl butoxide or malathion, inhibitors of P450s, to eliminate potential effects of enhanced herbicide degradation mediated by cytochrome P450 on growth. This enzyme family is usually the only one associated with metabolic resistance to ALS-inhibiting herbicides (Yu and Powles, 2014). Resistant and sensitive populations were collected, respectively, from the two neighboring provinces of Jiangsu and Anhui, in which arable fields generally have a similar herbicide application history over the past 20 years (Guo *et al.*, 2018). Multiple susceptible controls were used to minimize differences in genetic background between the ALS herbicide-resistant and various susceptible populations (Vila-Aiub *et al.*, 2015*a*). Independent comparison was made between each ALS herbicide-resistant population and all ALS herbicide-susceptible populations.

### ALS activity and kinetics associated with specific resistance mutations

Seed germination and seedling growth of *A. aequalis* individuals homozygous for each specific ALS resistance mutation (197-Tyr or 574-Leu) and susceptible individuals were performed according to Zhao *et al.* (2018). At the three- to four-leaf stage, young shoot tissues (about 4 g) were harvested from each population (at least 40 seedlings per harvest), snap-frozen in liquid N_2_, and stored at −80 °C. An *in vitro* ALS activity assay was conducted according to the method of Yu *et al.* (2004) with minor modifications. Frozen material was ground into a fine powder in liquid N_2_ and homogenized in 20 ml extraction buffer containing 100 mM potassium phosphate buffer (pH 7.5), 0.5 mM MgCl_2_, 0.5 mM thiamine pyrophosphate, 10 μM FAD, 10 mM sodium pyruvate, 10% (v/v) glycerol, 1 mM DTT, 0.5% polyvinylpyrrolidone, and 1 mM phenylmethylsulfonyl fluoride. The homogenate was centrifuged at 13 000 *g* for 15 min at 4 °C, after which an equal volume of saturated (NH_4_)_2_SO_4_ was added dropwise to the supernatant. The solution was kept on ice, slowly stirred for 10 min, and centrifuged at 13 000 *g* for 30 min at 4 °C. The pellet was dissolved in 2 ml of buffer solution containing 50 mM HEPES (pH 7.5), 200 mM sodium pyruvate, 20 mM MgCl_2_, 2 mM thiamine pyrophosphate, and 20 μM FAD. Soluble protein concentration of the enzyme extract was measured using the Bradford method (Bradford, 1976) and the sample was directly used for assay.

ALS activity was determined according to the method described by Yu *et al.* (2004). Firstly, 100 μl of enzyme extract and 100 μl of MM at different concentrations were mixed and incubated at 37 °C for 60 min. The reaction was stopped by adding 40 μl of 3 M H_2_SO_4_ followed by incubation at 60 °C for 15 min. Secondly, 190 μl of creatine solution (0.55% in deionized water) and 190 μl of α-naphthol solution (5.5% in 5 M NaOH) were added, after which the mixture was incubated at 60 °C for 15 min. Finally, the amount of acetoin formed was assayed colorimetrically at 530 nm on a microplate spectrophotometer (Epoch™, BioTek Instruments, Inc., Winooski, VT, USA). ALS activity was expressed as nmol acetoin formed per minute per milligram of protein employed for the assay. MM concentrations of 1×10^–6^, 1×10^–5^, 1×10^–4^, 1×10^–3^, 1×10^–2^, 1×10^–1^, 1, 10, 1×10^2^, and 1×10^3^ µM were used. Herbicide concentrations required to inhibit 50% of the ALS activity (*I*_50_) were calculated using non-linear regression analyses by fitting the data to the four-parameter logistic model *y*=*C*+(*D−C*)/[1+(*x*/*I*_50_)^*b*^] in SigmaPlot v14.0 (Systat Software, Inc., San Jose, CA, USA), where *C* is the lower limit, *D* is the upper limit, *b* is the slope at *I*_50_, and *y* is the response at herbicide dose *x*.

For ALS kinetic measurements, enzyme extracts were obtained using extraction and solution buffers without sodium pyruvate (Yu *et al.*, 2014). Herbicide MM was replaced with sodium pyruvate in a reaction system in which the final concentration of sodium pyruvate was 0.098, 0.195, 0.391, 0.781, 1.563, 3.125, 6.25, 12.50, 25, and 50 mM. *K*_m_ and *V*_max_ were calculated by fitting the data to the Michaelis–Menten equation *ν*=*V*_max_×*S*/(*K*_m_+*S*) in Prism 5.01 (GraphPad Software Inc., La Jolla, CA, USA), where *S* is the concentration of the substrate sodium pyruvate, and *ν* is the reaction velocity at the corresponding sodium pyruvate concentration. Two independent enzyme extracts were used for each assay set, and each assay contained three technical replicates. Means were separated using Dunnett’s *post hoc* test at the 5% level of probability.

### Growth assessment of plants without competition

Both classic and combined growth analyses of relative growth rate (RGR) and its components including net assimilation rate (NAR) and leaf area ratio (LAR) were made in *A. aequalis* plants without competition (Hunt, 1982; Poorter, 1989; Hoffmann and Poorter, 2002). Seeds of the homozygous ALS herbicide-resistant mutants (197-Tyr and 574-Leu) and susceptible ALS-wild-type genotypes (S1 to S3) were germinated as described above. Individual *A. aequalis* seedlings at 2 cm height were transplanted into individual 9 cm-diameter plastic pots containing 50% sandy loam and 50% nursing medium. All plants were grown in a controlled greenhouse (25/15 °C day/night, natural light, ~75% relative humidity) and watered regularly. Experiments were arranged in a completely randomized design with all pots regularly rearranged to randomize any environmental differences within the greenhouse.

For classic growth analysis, 10 plants were harvested for each population at 28 and 48 d after transplanting (DAT), and their aboveground biomass (dry weight) was respectively recorded. Leaf area per plant was determined using a handheld leaf area meter (YMJ series, TOP Holding Co., Ltd, Zhejiang, China). The net photosynthetic rate (*P*_n_) was measured on the second fully expanded leaf of each plant using a portable photosynthesis system (Ciras-3, PP Systems, Hitchin, UK). During the measurement, the average leaf temperature, relative air humidity, photon flux density, and ambient CO_2_ concentration were 30 °C, ~75%, 1200 μmol m^−2^ s^−1^, and 370–400 ppm, respectively. RGR and its components were calculated using an unbiased formula proposed by Hoffmann and Poorter (2002). One-way analysis of variance (ANOVA) coupled with Dunnett’s *post hoc* test (α=5%) was performed to determine pairwise differences in growth estimates (RGR, NAR, and LAR) and *P*_n_ values between each homozygous ALS herbicide-resistant population and the susceptible ALS-wild-type reference populations.

For combined growth analysis, aboveground shoots were harvested at 32, 36, 40, 44, 48, 52, and 56 DAT. Twenty plants were selected for each harvest. Mean values of RGR, NAR, and LAR estimated for each harvest interval were fitted to the splined cubic polynomial model *y*=*y*_0_+*ax*+*bx*^2^+*cx*^3^ in SigmaPlot v14.0, where *y* represents the RGR, NAR, or LAR of the plant, *x* is time, *y*_0_ is the *y* value when *x*=0, and *a*, *b*, and *c* are the rates of increase in growth traits at different harvest times (Hunt and Evans, 1980; Hunt, 1982).

### Growth assessment of plants under competition

A target-neighborhood design (Fig. 5A) was used to evaluate competitive responses of homozygous mutants and susceptible plants growing in competition with wheat (*Triticum aestivum* L., hybrid Jimai 22) (Goldberg, 1990; Li *et al.*, 2013). After seed germination, 2 cm-tall seedlings of both wheat and *A. aequalis* were simultaneously transplanted into 20.5×40 cm (diameter×height) plastic pots containing 50% sandy loam and 50% nursing medium. Four *A. aequalis* plants per pot were subjected to competition from neighboring wheat at densities of 0, 30, 60, 120, and 240 plants per m^2^ (Vila-Aiub *et al.*, 2009*a*; Du *et al.*, 2019). Pots were placed outdoors during the normal *A. aequalis* growing season and were regularly rearranged to randomize any environmental differences. All pots were kept well watered at all times, and a slow-release fertilizer (DeWoDuo, Hengshui, China; 10 g per pot; N+P_2_O_5_+K_2_O≥42%) was applied during the tillering phase. After 2 months of growth, aboveground biomass of both *A. aequalis* and wheat was determined. Leaf area for individual *A. aequalis* plant was also measured as described above. Experiments were arranged in a completely randomized design and each treatment consisted of six replicates.

Per plant and unit-size competitive responses of the target plants to increasing density and biomass of neighboring wheat plants were analysed using the hyperbolic non-linear model *y*=*a*/(1+*bx*), where *y* represents the biomass or leaf area of *A. aequalis* at wheat density or biomass *x*, *a* is the biomass or leaf area of *A. aequalis* in the absence of competitors (*x*=0), and *b* is the slope of the regression. Steeper slopes denote weaker competitive responses.

## Results

### Effects of ALS herbicide resistance mutations on ALS activity and kinetics

**Fig. 2.**
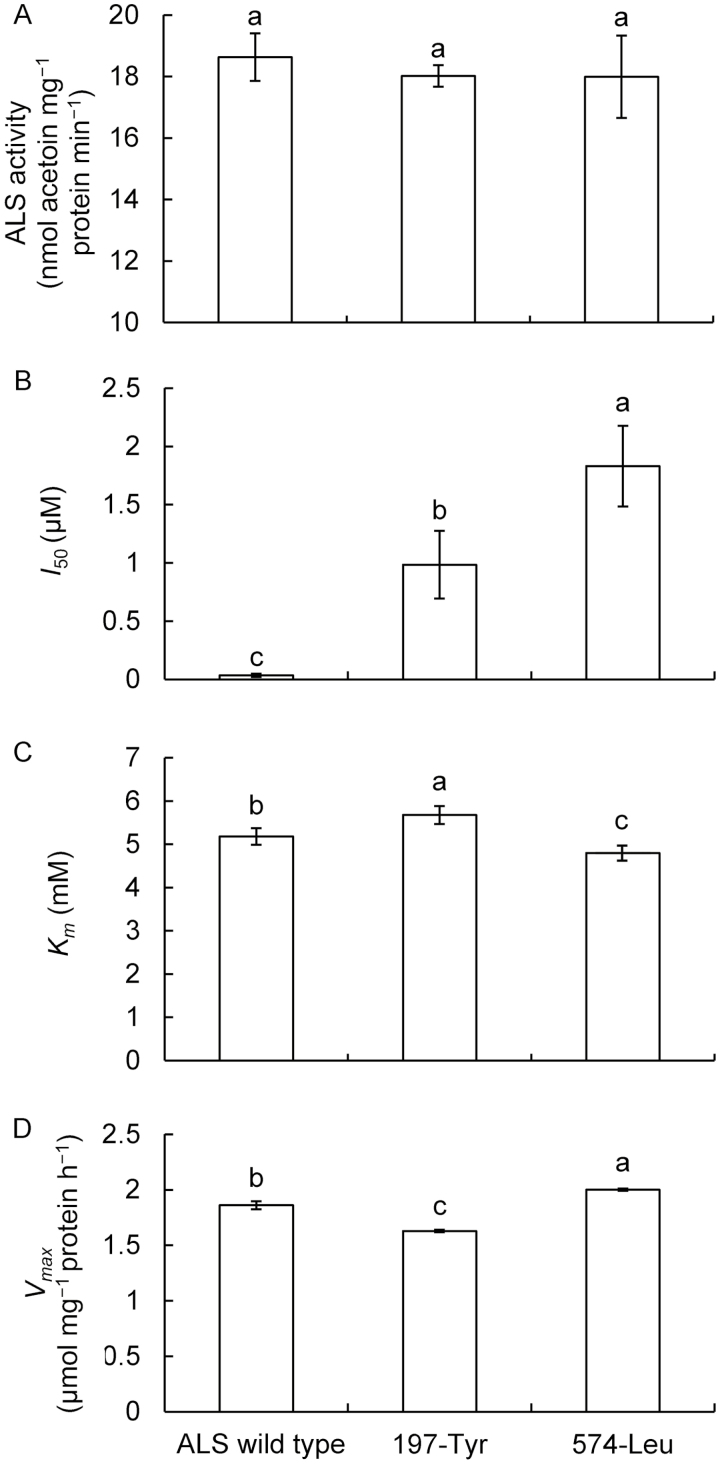
Characteristics of ALS measured from partially purified enzyme extracts from three- to four-leaf-stage ALS herbicide-susceptible (ALS wild type), and homozygous resistant (RR) mutant (197-Tyr and 574-Leu) *A. aequalis* plants. Comparisons were made between populations carrying each specific *ALS* gene mutation and ALS-wild-type populations. Different letters indicate significant differences in mean estimates according to Dunnett’s test (α=0.05).

No significant differences in estimated ALS activity and kinetics were found among three herbicide-susceptible populations (*P*>0.05); therefore, a mean value was calculated for all susceptible populations and used as a reference estimate for further comparisons. In the absence of MM treatment, the extractable ALS activity in *A. aequalis* plants homozygous for each mutation was not significantly different from that in ALS-wild-type plants (Fig. 2A). After MM treatment, wild-type ALS was strongly inhibited with an *I*_50_ value of 0.0355 μM (Fig. 2B). By contrast, the sensitivity of mutated ALS to MM was greatly decreased, with *I*_50_ values 27.7- to 51.6-fold greater than that of wild-type ALS. The 574-Leu mutant ALS was the least sensitive to MM, followed by the 197-Tyr mutant ALS (Fig. 2B).

We determined the ALS substrate affinity (*K*_m_) for pyruvate for each resistance mutation and compared this with the wild-type ALS controls. The two mutations showed contrary effects on ALS substrate affinity: the 197-Tyr mutation significantly increased the *K*_m_ value for pyruvate, whereas the 574-Leu mutation slightly reduced the *K*_m_ (Fig. 2C). Calculated *V*_max_ also revealed that maximum reaction velocity was decreased in the 197-Tyr mutants but increased in the 574-Leu mutants compared with wild-type ALS controls (Fig. 2D).

### Examination of resistance costs

#### Growth of isolated plants without competition

**Fig. 3.**
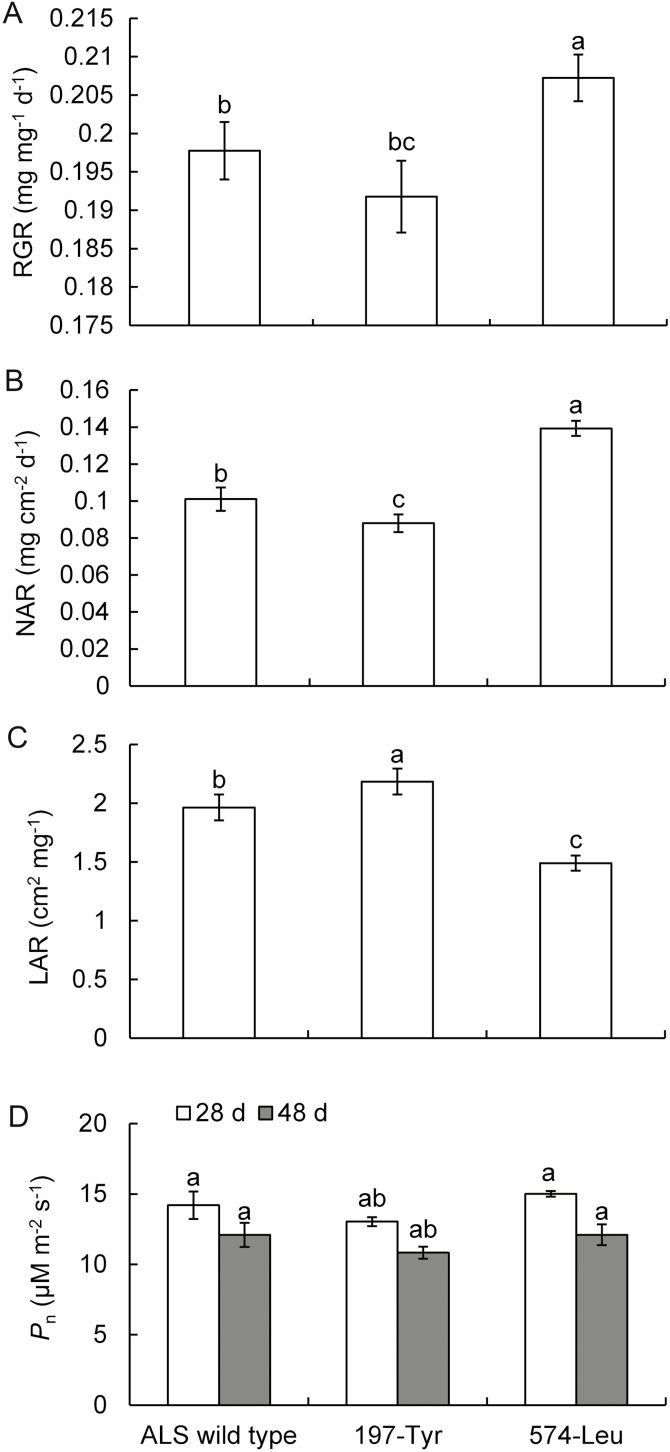
Mean estimates of RGR and its components NAR and LAR associated with *A. aequalis* genotypes containing wild-type ALS and specific homozygous (RR) resistance mutants (197-Tyr or 574-Leu). Growth was estimated in isolated plants in the absence of herbicide treatment for a period of 20 d from 28 d after transplanting. Comparisons were made between populations carrying each specific *ALS* gene mutation and ALS-wild-type populations. Different letters indicate significant differences in mean estimates according to Dunnett’s test (α=0.05).

Estimated growth parameters did not differ among the three susceptible populations (*P*>0.05), and thus a mean value was calculated for all susceptible populations and used as a reference estimate for further comparisons. Homozygous 197-Tyr mutants exhibited significantly reduced RGR and NAR growth parameters and *P*_n_ values compared with ALS-wild-type plants (Fig. 3A, B, D). In contrast, plants homozygous for the 574-Leu mutation exhibited significantly increased RGR, driven by an increase in NAR but not LAR, representing a resistance advantage of 4.6% associated with RGR (Fig. 3C). At 28 and 48 DAT, homozygous 574-Leu mutant plants exhibited similar *P*_n_ parameters to those of ALS-wild-type plants, which were significantly higher than those of 197-Tyr mutant plants (Fig. 3D).

**Fig. 4.**
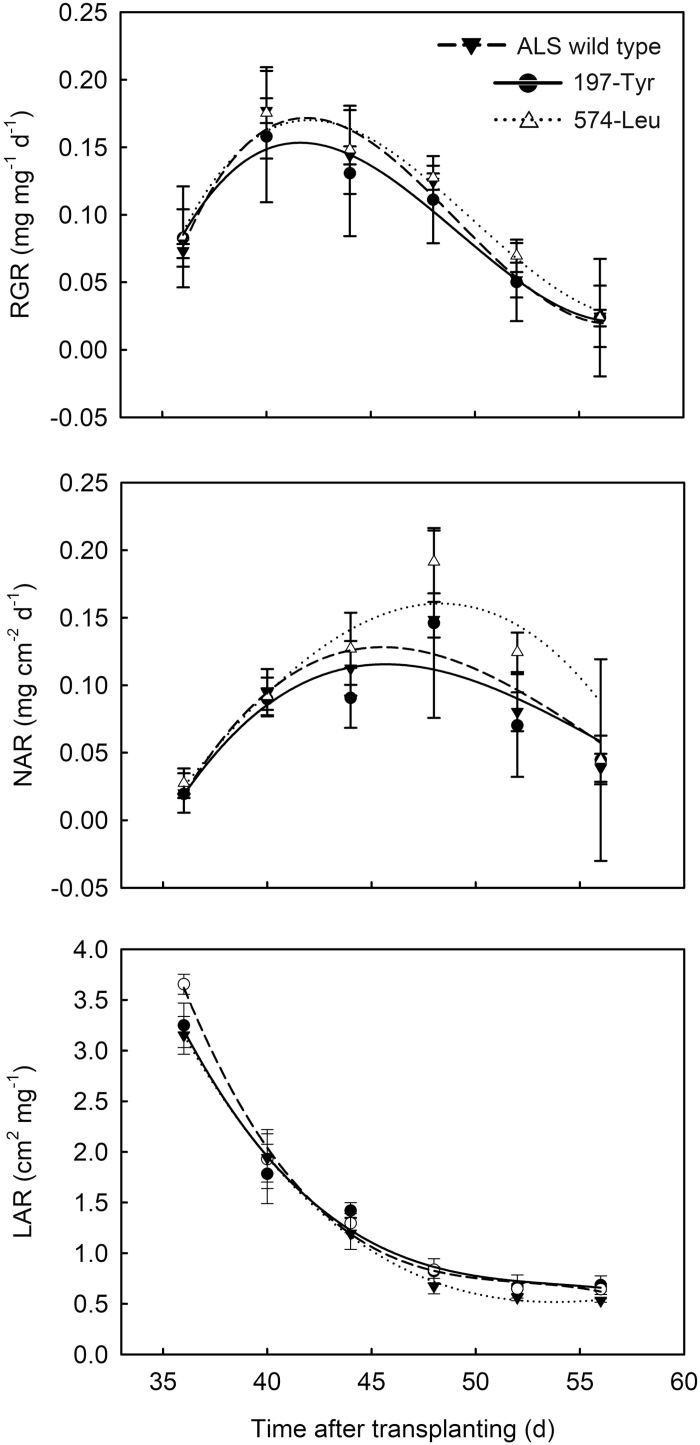
Changes in the mean estimates of RGR, NAR, and LAR over time for each *A. aequalis* genotype: wild-type ALS (▼) and specific homozygous (RR) 197-Tyr (●) or 574-Leu (△) resistance mutants.

We also conducted a combined growth analysis for a period of 24 d starting at 32 DAT. During this growth period, RGR and NAR values of all plants peaked at 40–44 and 44–52 DAT, respectively, and then decreased as time progressed (Fig. 4). Comparisons between homozygous mutants and ALS-wild-type plants revealed that the RGR and NAR of 197-Tyr mutant were significantly reduced by 9.7% and 6.9%, respectively. By contrast, the RGR and NAR of the 574-Leu plants increased by 5.5% and 22.6%, respectively.

#### Growth of plants under competition

**Fig. 5.**
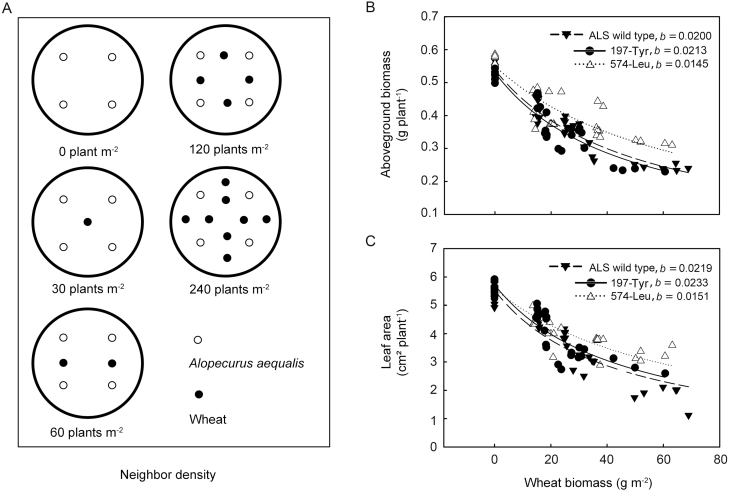
(A) Overview of experimental design (target-neighborhood model) to assess plant responses associated with ALS herbicide-resistant (197-Tyr and 574-Leu) and susceptible (ALS wild type; S1, S2, and S3) alleles of *A. aequalis* (○) to competition with wheat (●) at the vegetative plant stage. (B, C) Variations in aboveground vegetative biomass (B) and leaf area (C) of ALS-wild-type (▼) and ALS inhibitor-resistant 197-Tyr (●) and 574-Leu (△) mutant individuals with increasing biomass of neighboring wheat plants. Data are mean biomass of *A. aequalis* individuals from one pot. For each neighbor density tested, two replicates from each of the three susceptible populations were randomly selected. Comparison of regression slopes (*b* parameter) determines hierarchies in per unit-size competitive responses of target plants to neighboring wheat plants.

Increased density (per plant response) and overall biomass (per unit-size response) of competing wheat plants significantly affected the aerial biomass and leaf area of target *A. aequalis* plants. Because per plant and per unit-size based competitive responses of target plants were similar, only the latter are shown. Slopes (*b* parameter) estimated after regression revealed bigger reductions in both aerial biomass and leaf area of 197-Tyr plants compared with ALS-wild-type plants (Fig. 5B, C). By contrast, 574-Leu mutants produced concomitantly more biomass than ALS-wild-type target plants with increasing wheat competition (Fig. 5B). These increases in target plant biomass were accompanied by a smaller reduction in leaf area compared with that in ALS-wild-type individuals (Fig. 5C).

## Discussion

Diverse gene mutations conferring TSR to ALS inhibitors have been identified in arable weed species (Powles and Yu, 2010; Délye *et al.*, 2013; Yu and Powles, 2014; Rey-Caballero *et al.*, 2017; Tranel *et al.*, 2019). Generally, such mutations in weeds may adversely affect their growth and fitness, accounting for the likely persistence or fixation of novel resistance mutations in untreated weed populations or populations in which selection has been discontinued (Jasieniuk *et al.*, 1996; Vila-Aiub *et al.*, 2015*b*). For annual species, changes in growth rate and plant size often correlate positively with changes in reproductive traits (Bazzaz *et al.*, 1987; Weiner, 2004; Vila-Aiub *et al.*, 2009*a*). In the current study, we selected *A. aequalis* plants carrying homozygous *ALS1* gene mutations resulting in 197-Tyr or 574-Leu amino acid substitutions, and assessed various growth traits defining the overall population fitness associated with these two specific ALS mutations. Correlations between these fitness components and the impact of the two mutations on ALS activity and kinetics were also examined. Because no ACCase-wild-type individuals were isolated from the original AH-12 population, both resistant populations simultaneously carried a homozygous mutation in the *ACCase* gene producing a 2078-Gly substitution. The ALS-wild-type populations S1, S2, and S3 possessing the homozygous *ACCase* mutation causing 2078-Gly substitution were used as controls to minimize differences in genetic background between the ALS herbicide-resistant and various susceptible populations (Vila-Aiub et al., 2009*b*, 2011). Our results revealed that (i) both the 197-Tyr and 574-Leu resistance mutations endowed weed plants with resistance to ALS inhibitors without significant changes in extractable ALS activity; (ii) the two mutations had contrary effects on substrate binding (*K*_m_) for pyruvate and the maximum reaction velocity (*V*_max_) of ALS; (iii) catalytic capacity associated with different mutations was correlated with the cost of resistance.

### ALS 197-Tyr mutation: impact on ALS kinetics and resistance costs

*Alopecurus aequalis* plants homozygous for the 197-Tyr mutation showed slightly reduced RGR-NAR growth parameters without competition, suggesting a negative association with the plant’s efficiency in capturing light, assimilating CO_2_, and/or storing photoassimilates (Vila-Aiub *et al.*, 2015*b*). This is consistent with the lower photosynthetic rates of 197-Tyr mutants compared with ALS-wild-type plants. Under competition with wheat, plants with the 197-Tyr mutation displayed a consistently weak competitive response when compared with plants with the wild-type ALS. Because these responses were consistent with increasing wheat density and aboveground biomass, the weak competitive response of 197-Tyr mutants was most likely contributed by the specific *ALS* gene mutation rather than reduced plant size (Goldberg and Scheiner, 2001). Under competitive conditions, light becomes the most limiting resource (Vila-Aiub *et al.*, 2015*b*); thus, *A. aequalis* plants exhibiting reduced NAR are expected to make inefficient use of radiation.

*ALS* gene mutations conferring herbicide resistance may lead to unchanged or reduced ALS activity. However, Yu *et al.* (2010) reported that most *Lolium rigidum* plants homozygous for different resistance-conferring *ALS* gene mutations showed significantly higher extractable ALS activity. ALS-inhibiting herbicides do not bind at the active site, but bind within the substrate-access channel, thereby blocking substrate access to the active site (Li *et al.*, 2019; Liu *et al.*, 2019). Therefore, these resistance mutations do not drastically change pyruvate binding and the mutated ALS still functions normally (McCourt *et al.*, 2006). Similarly, for a number of ALS resistance mutations, unchanged, increased, or reduced *K*_m_ values have been observed in different plant species (reviewed by Yu *et al.*, 2010). These contradictory reports again indicate that the impact of each specific mutation needs to be evaluated on a case-by-case/species-by-species basis. In the present study, the 197-Tyr mutation did not change the extractable ALS activity of *A. aequalis*, but slightly decreased the substrate affinity (increased *K*_m_ value) and velocity (*V*_max_) of ALS to catalyse the formation of 2-acetolactate at the expense of pyruvate. These minor reductions may lead to a shortage of branched-chain amino acids available for rapid growth, correlating with the impaired growth responses observed in plants homozygous for the ALS 197-Tyr mutation.

### ALS 574-Leu mutation: detectable resistance advantages

*Alopecurus aequalis* homozygous 574-Leu resistance mutants showed higher efficiency in capturing light, assimilating CO_2_, and/or storing photoassimilates, evidenced as increased RGR-NAR growth parameters compared with plants with the ALS-wild-type ALS. Under competition, plants with the 574-Leu mutation also showed stronger competitive responses for resources compared with ALS-wild-type plants. We observed a positive correlation between *V*_max_ associated with Trp-574 mutation and growth responses. Resistance advantages associated with the 574-Leu mutation in *A. aequalis* likely result from the slightly increased maximum velocity (*V*_max_), resulting in fast product biosynthesis. In *L. rigidum*, 574-Leu mutation also significantly increases the *V*_max_ of ALS but has no significant effects on plant growth (Yu *et al.*, 2010). Our results are contradictory to those of Tardif *et al.* (2006) showing that the 574-Leu mutation has considerable pleiotropic effects on early growth and development of the annual weed *Amaranthus powellii*, greatly reducing fitness under competitive conditions; however, this is reasonable because specific effects of resistance mutations on ALS greatly depend on species (Yu *et al.*, 2010).

### Prediction of the evolutionary dynamics of ALS herbicide resistance

Negligible resistance costs or significant fitness advantages might manifest in herbicide-resistant weed populations without herbicide selection, while plant protection under herbicide selection would favor the rapid evolution and fixation of novel herbicide TSR alleles (Vila-Aiub *et al.*, 2009*a*). High levels of resistance to several ALS-inhibiting herbicides conferred by the 197-Tyr and 574-Leu mutations have been characterized in various species (Délye *et al.*, 2009; Yu *et al.*, 2012; Hamouzová *et al.*, 2014; Zhao *et al.*, 2019). For *A. aequalis*, decreased susceptibility of ALS to MM in both 197-Tyr and 574-Leu mutants supports this (Fig. 2B). Therefore, under ALS-inhibiting herbicide selection, the population frequency of both the 197-Tyr and 574-Leu mutations is expected to increase.

In the absence of herbicide selection, a significant fitness advantage suggests no apparent constraints for the ALS 574-Leu resistance allele to be maintained and fixed in populations. A large-scale investigation on the resistance status of *A. aequalis* in eastern China supports this; a number of homozygous 574-Leu mutants were collected under field conditions, some from fields out of cultivation (Guo *et al.*, 2018). By contrast, slight resistance costs suggest some environmental limits for the ALS 197-Tyr resistance allele to persist once herbicide selection is eliminated. To date, only two *A. aequalis* populations—one reported by Zhao *et al.* (2019) and the other reported here—with heterozygous 197-Tyr mutations have been identified from dozens of populations collected across eastern China (unpublished data). The rarity of 197-Tyr may be partially due to both the low frequency of double mutation at the first two nucleotides (CC) of a Pro codon (Sada and Uchino, 2017) and the result of the associated impaired plant growth (Figs 2–4). Thus, if herbicide application is discontinued, the 574-Leu mutation is more likely to persist than the 197-Tyr mutation.

Notably, all the populations used in the current study simultaneously possessed a homozygous *ACCase* gene mutation producing 2078-Gly. Fitness costs associated with this amino acid substitution have been reported in different grass species such as *L. rigidum*, *Beckmannia syzigachne*, and *Alopecurus myosuroides* (Vila-Aiub *et al.*, 2015*b*; Menchari *et al.*, 2008; Du *et al.*, 2019). This suggests that the *ACCase* gene mutation causing 2078-Gly should have deleterious effects in *A. aequalis*, making the prediction of evolutionary dynamics more complex for the resistant populations used in this study. If the *ACCase* gene mutation causing 2078-Gly substitution indeed confers a fitness cost, population frequencies of AH-12 (197-Tyr) would be expected to gradually reduce when herbicide application is discontinued. For the AH-28 (574-Leu) population, further growth assessments including fully susceptible populations need to be conducted to evaluate the contribution of this mutation to fitness costs.
